# Isosorbide Diesters: Mechanistic Insights and Therapeutic Applications in Skin and Neuroinflammatory Disorders

**DOI:** 10.3390/ijms262411855

**Published:** 2025-12-09

**Authors:** Ratan K. Chaudhuri, Thomas A. Meyer

**Affiliations:** 1 CuralysMD Dermaceuticals & Sytheon (Now Part of Hallstar), 10 Waterview Blvd, Parsippany, NJ 07054, USA; 2 Meyer Sun and Skin Care Consultancy, Memphis, TN 38139, USA

**Keywords:** isosorbide fatty acid diesters, epidermal barrier repair, skin hydration, anti-inflammatory activity, atopic dermatitis, skin microbiome modulation, psoriasis, multiple sclerosis

## Abstract

Isosorbide fatty acid diesters constitute a novel class of bioactive compounds with emerging therapeutic applications in inflammatory and barrier-compromised disorders. Among them, isosorbide dicaprylate (IDC) and isosorbide di-linoleate/oleate (IDL) synergistically strengthen epidermal barrier integrity, enhance stratum corneum hydration, regulate keratinocyte differentiation, suppress proinflammatory signaling, and beneficially modulate the skin microbiome. Randomized, double-blind clinical trials in both pediatric and adult populations with atopic dermatitis (AD) demonstrate that topical IDC + IDL formulations significantly reduce pruritus, corticosteroid dependence, and *Staphylococcus aureus* colonization while improving sleep quality, disease severity scores, and overall quality of life. Extending applications within and even beyond dermatology, isosorbide dimethyl fumarate (IDMF)—a next-generation fumarate derivative designed to mitigate sensitization risk—exhibits potent anti-inflammatory and antioxidant activities through NRF2 activation and NF-κB/IRF1 suppression. Preclinical studies in psoriasis and neuroinflammatory models, including multiple sclerosis, reveal robust modulation of oxidative stress and immune pathways with improved safety and mechanistic precision compared to conventional fumarates, although its systemic use remains exploratory and requires clinical validation. Collectively, isosorbide diesters emerge as multifunctional therapeutic agents offering barrier repair, immune modulation, and inflammation control, representing promising alternatives to corticosteroids and systemic immunosuppressants across dermatologic and systemic inflammatory disorders.

## 1. Introduction

An ever-growing body of research continues to reinforce the connection between integrity of the barrier function (BF) and skin’s overall health and beauty [1,2]. Disruption of BF constitutes a prominent feature in recurring inflammatory skin diseases, including atopic dermatitis [3] and psoriasis [4] as well as in xerosis [5] and aged skin [6]. BF dysfunction robs skin of its natural beauty and is associated with many common skin afflictions, including acne [7], rosacea [8] and fungal infections [9]. More recently, emerging research indicates that, beyond natural aging, a compromised skin barrier may contribute to systemic inflammation and chronic disorders that commonly accompany aging in the elderly (atherosclerosis, type II diabetes, osteoporosis and cognitive decline) [10,11,12]. Research also suggests that a compromised skin barrier can trigger systemic immune responses [13]. Understanding the interplay between the integrity of skin’s barrier function and systemic health could uncover new intervention strategies to promote healthier aging.

Many aspects of skin’s BF are associated with the stratum corneum (SC), the outermost layer of the epidermis, whose architecture and functions have been extensively reviewed elsewhere [2,14]. Briefly, these reviews describe the structure of the stratum corneum (SC) as resembling a brick wall, where the “bricks” are flattened, anucleated corneocytes packed with keratin filaments and water-binding molecules collectively known as natural moisturizing factor (NMF). Their plasma membranes are replaced by an insoluble scaffold of cross-linked proteins, externally coated with a hydrophobic lipid layer together forming the corneocyte envelope. For extra strength and reinforcement, adjacent corneocytes are also attached to one another via corneodesmosomes, which have been likened to use of iron rods in brick walls [15]. The mortar between the bricks comprises planar sheets of multilamellar lipid bilayers composed principally of ceramides, cholesterol and free fatty acids. Together these key physical structures help regulate two of BF’s most hallmark functions, namely prevention of excessive water loss and retardation of penetration of harmful irritants and pathogens. Additionally, intact SCs also possess antimicrobial, antioxidant and acidified surface barriers along with an immunological barrier principally associated with Langerhans cells that reside just beneath the SC [2].

SC is also in a constant state of flux as it is continuously being regenerated from underneath through differentiation [16]. In this process, new cells proliferated in the basal layer are gradually pushed up and outward toward the surface. Along this journey, cells undergo tremendous biochemical and morphological alterations that eventually give rise to flattened corneocytes and the specialized architecture of the SC [16]. To maintain homeostasis and to balance the generation of new cells at the bottom, skin constantly sheds old cells at the surface through desquamation [17]. In normal skin, desquamation occurs unnoticed as skin cells are shed individually, while in xerosis with disruption in desquamation, cells shed in clumps and appear as white, flaky skin [5]. In AD cells desquamate prematurely [18], whereas in psoriasis desquamation though accelerated cannot keep pace with the rapid increase in cell turnover rates (~7 times faster than normal skin), which results in buildup of cells at the surface and formation of scaly, thickened plaques characteristic of the disease [4].

Thus, BF comprises a plurality of components and functions. Research has further established that the various components of BF do not operate independently of one another but rather function interdependently, with dysfunction in one barrier potentially affecting the function of other barriers [3,18]. As the interface with the external world, skin as a whole but SC in particular as the outermost layer of epidermis is under constant assault by a myriad of environmental stressors, including exposures to sun, aerial pollution (microparticulate matter, ozone), low ambient temperatures and relative humidities, chemical irritants from daily hygiene products, allergens, psychological stress and even house dust mites as a recognized stressor in AD skin [18,19,20]. All these various stressors can act individually or in concert to induce damage within SC.

The damage to SC caused by environmental stressors produces defects in BF that promote increased rates of transepidermal water loss (TEWL), decreased levels of SC hydration, elevated skin surface pH, and makes skin vulnerable to penetration by exogenous irritants and pathogens, all of which foster cutaneous inflammation [18,19,20]. Inflammation delays barrier recovery and hampers terminal differentiation, which continues to perpetuate BF dysfunction even further [21,22]. Epidermal dysfunction has also been linked to a condition termed inflammaging, characterized by chronic subclinical inflammation that continually promotes skin aging [23], exacerbates inflammatory diseases [23,24,25] and contributes to systemic aging [6,10,11,12].

With its high vulnerability to damage from environmental stressors, skin requires constant care and attention even though the epidermis continually regenerates itself. With increased and emerging elucidation of BF components and how they interact, attention has focused recently on optimizing topical moisturizers (or emollients) for their ability to help repair, maintain and even strengthen BF daily [22,26,27,28,29]. This is especially relevant in long-term management of mild to moderate AD where even normal-looking, non-lesional areas of skin still display abnormal BF properties and signs of subclinical inflammation compared with normal, healthy skin [30]. For these reasons, global treatment guidelines for AD consistently recommend daily and frequent use of moisturizers as a key component of AD management [31]. Restoration of the barrier function also makes skin more resilient to irritants and allergens [22]. In addition, bolstering BF properties with daily applications of effective moisturizers constitutes an essential part of antiaging strategies in normal skin aimed at preserving skin’s more youthful appearance and beauty for as long as possible throughout life [32].

Over recent years, however, the efficacy of moisturizers has come under increased scrutiny [33,34,35]. This is especially true in skin with already weakened skin barriers (as in AD, sensitive skin or other skin disorders) where multiple studies support that moisturizers can exert very different effects on the barrier function depending on their formula composition [28,36,37,38,39,40]. These studies emphasis that moisturizer effects can encompass (1) further elicitation of disruption to the barrier function [36,40], (2) transient improvements for short periods of relief after application but with no corrections to skin’s underlying barrier defects [28,39] or (3) measurable enhancements and strengthening of barrier function for longer-term benefits [38,39].

Several key formulation strategies associated with BF enhancements and strengthening have been identified, including deployment of buffered acids to adjust skin surface acidity within pH 4–6 [41,42]; humectants to draw and hold water [42]; lipids to replenish, repair and fortify lipid bilayers [26]; occlusive films to retard surface water loss [43]; and many other types of specialized or natural ingredients with anti-pruritic or anti-inflammatory activity that function through alternative mechanisms within skin to enhance BF capacity. This includes, for example, ingredients that when incorporated into cosmetic formulations exhibit ability to favorably affect expression of genes involved in keratinocyte differentiation and formation of the barrier function [44,45]. Taken together, manipulation of these formulation strategies can help enhance hydration, reduce TEWL, repair defects in BF and create an unfavorable environment for pathogenic organisms (*Staphylococcus aureus*), thereby helping to maintain integrity of skin’s BF in a proactive manner that is more akin to normal, healthy skin.

**Figure 1:**
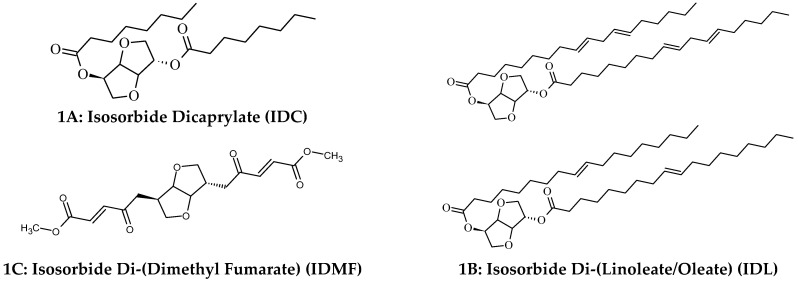
Structures of isosorbide diesters.

This overview serves to highlight the disparate properties of a novel class of plant-derived fatty acid di-esters based on isosorbide (Figure 1) for utility in dermatology and systemic inflammatory disorders, such as multiple sclerosis (MS). These include isosorbide dicaprylate (IDC, **1A**), isosorbide di-(linoleate/oleate) (IDL, **1B**) and isosorbide di-(methyl fumarate) (IDMF, **1C**) for topical skin care applications, including long-lasting hydration, anti-aging benefits, management of AD and treatment of psoriasis, as well as IDMF for systemic treatment of relapsing-remitting multiple sclerosis. For skincare, these fatty acid di-esters represent specialized ingredients that function predominately within skin through mechanisms other than their direct ability to affect acidification, humectancy, occlusivity or serve as lipid replacement therapy and as such constitute new adjuvants to incorporate into new and existing formulations for enhanced benefits. Herein, we highlight key insights into the potential mechanisms of action derived from gene expression profiling and targeted protein quantification alongside evidence of their efficacy in clinical studies.

## 2. Methodology

This manuscript was developed as a narrative review rather than a systematic review. A systematic review would require a registered protocol, comprehensive database searches based on predefined inclusion and exclusion criteria, and adherence to methodological procedures consistent with PRISMA guidelines. These steps were not within the intended scope of the present work.

Instead, this review provides a targeted and integrative overview of current evidence regarding the benefits of isosorbide diesters in skin care—particularly in the context of atopic dermatitis, xerosis, and ichthyosis—and their potential as novel fumarate-based therapeutics for psoriasis and multiple sclerosis (MS). Relevant literature was identified through focused searches in Google Scholar, SCOPUS, and PubMed, and all pertinent studies are cited accordingly. Reference selection was guided by scientific relevance to the topics addressed rather than by systematic criteria. Maintaining the narrative-review format ensures that the manuscript accurately reflects its intended purpose and scope and provides an accessible synthesis of the available evidence for readers.

## 3. Dermatological Applications

### 3.1. Cutaneous Metabolism of Di-Fatty Acid Isosorbide Esters

Previous studies have shown that di-fatty acid isosorbide esters within skin are highly susceptible to hydrolysis by carboxylesterase 2 (CES2), which is the abundant form of the enzyme expressed within keratinocytes (KCs) [46]. Model studies on isosorbide-based prodrugs have further demonstrated that while the 2-ester group underwent rapid hydrolysis, little hydrolysis of the 5-ester group occurred [47]. Accordingly, it could be expected that the action of CES2 on the isosorbide-based esters reviewed here would yield a mixture of hydrolysis products that would broadly include 5-monoester metabolites plus liberated fatty acids (linoleic and oleic acids), in addition to any remaining intact isosorbide di-ester. In this regard, the action of CES2 toward isosorbide di-esters differentiates it from that toward linear di-esters or monoesters, which appear to be hydrolyzed extensively to liberate the free fatty acid moieties. This has repercussions for IDL and IDMF whose fatty acid moieties are known to possess biological activity and thus could exert differential effects on gene expression profiles compared with the metabolites or intact molecule of their isosorbide-based esters. These will be discussed in greater detail in the sections below.

### 3.2. IDC and IDL Promote Hydration and Restoration of Disrupted Barrier Function

The ability of BF to manage water loss and ultimately water content of SC exerts critical influence on appearance and health of skin. Proper hydration within the SC (1) plasticizes the tissue keeping it soft, pliable and elastic, which enhances radiance and glow plus minimizes the appearance of fine lines and wrinkles to optimize appearance and beauty; (2) affords physical protection against mechanical shear forces; (3) sustains optimal function of key hydrolytic enzymes in the SC needed to form a mature SC with competent BF, including intercellular lipases that convert precursor lipids into final lipid components required for creation of lipid bilayers, intracellular proteases that process filaggrin into natural moisturizing factors (NMF) and intercellular serine proteases that degrade corneodesmosomes in a highly controlled manner to facilitate the process of desquamation and (4) dampens inflammation [2,5,6,18,48]. More recently, SC hydration but not skin surface pH or TEWL (for the most part) was found to correlate inversely with serum levels of proinflammatory cytokines in aged individuals (>65), suggesting that reduced SC hydration may even play a role in development of systemic disorders associated with aging [11].

#### 3.2.1. Clinical Studies Confirm That IDC Enhances, Prolongs Skin Hydration and Strengthens the Barrier Function

A cohort of clinical studies [49] has illustrated compelling evidence that topical application of IDC not only enhances but also prolongs hydration levels in skin after cessation of product use. On moderately dry skin, in the absence of other humectants, twice daily application over 14 days of a lotion containing 2% IDC was 9-fold more effective than its placebo lotion in improving skin hydration and importantly after a 4-day regression period the IDC lotion still maintained 57% of its original hydration boost. On severely dry skin, twice daily application of the IDC lotion over 7 days elevated hydration levels by 45% from baseline and by comparison was 3 times more effective than the same placebo lotion containing 2% glycerol. Importantly, co-formulation of 2% IDC with 2% glycerol demonstrated a synergistic effect, enhancing hydration levels beyond the additive effects of IDC or glycerol alone. Notably, the three-fold increase in hydration observed with the combined formulation after 14 days of treatment persisted even after a 4-day regression period compared with glycerol alone. These comparisons are particularly meaningful given that glycerol is an endogenous humectant [50] and is widely recognized as an effective hydrating agent in topical moisturizers [48]. Furthermore, due to their distinct molecular weights (398 g/mol for IDC vs. 92 g/mol for glycerol), the application of approximately equal volumes of the lotions in the cited clinical studies delivers nearly four times as many glycerol molecules as IDC to the skin. Since glycerol primarily functions as a humectant to bind water, these findings suggest that IDC enhances hydration within the SC through mechanisms beyond simple passive water binding.

In addition to its ability to boost hydration, the application of the 2% IDC lotion also displayed clinical ability to reduce TEWL significantly in severely dry skin relative to its vehicle control [49]. This suggests that at least one mechanism other than as a humectant by which IDC boosts hydration is through enhancement of barrier recovery or repair to help restrict water loss to the environment. Over regular periods of application, IDC may also help maintain competency of BF in the face of daily environmental insults that are known to damage and weaken skin’s barrier function [19]. This is important since ultimately the level of hydration within skin depends upon its balance between water retention versus water loss through transepidermal evaporation (i.e., TEWL).

#### 3.2.2. Mechanistic Insights into IDC’s Ability to Promote Epidermal Skin Hydration

Within the architecture of the SC, studies show that water resides predominantly within corneocytes [2,3] and that under normal conditions of hydration free water content within the fluid domains of the intercellular lipid lamellae does not exist [51,52]. The fact that the lipid lamellae do not swell even under normal conditions of higher hydration is further testament to their resilience in maintaining SC’s main permeability functions, namely as a barrier to excessive water loss as well as a barrier to penetration by external pathogens and irritants [51,52].

Within corneocytes, water is associated with the presence of NMF, a group of hygroscopic molecules that bind and retain water [2,53]. The main constituents of the NMF comprise amino acids, lactic acid, sodium pyrrolidone carboxylic acid (PCA) and urea and are derived from the breakdown of the protein filaggrin [2,53]. NMF draws water to the SC from underlying viable cell layers as well as from the outside environment. In fact, as a responsive organ, skin in part regulates NMF levels in response to environmental conditions, with low humidity climates stimulating more while high humidity climates stimulating less NMF production [18]. Corneocytes possess the capacity to swell by as much as 50% by binding water, mostly in vertical as opposed to the horizontal direction [54]. This, in turn, plumps corneocytes which further helps to restrict paracellular penetration of allergens and irritants (thus strengthening the permeability barrier) and to diminish the appearance of fine lines and wrinkles as well as enhance elasticity and mechanical strength for cosmetic improvements.

In addition to the hygroscopic properties of NMF inside corneocytes, other pathways are well known to play important roles in skin hydration. In general, these encompass hydration status of viable cellular layers (including epidermis and dermis) beneath the SC as well as key components of BF that restrict water loss from the skin to the environment, including the orderly lamellar arrangement of intercellular lipids and the presence of tight junctions within the granular layer [48]. Accordingly, gene expression data has revealed that IDC significantly upregulates genes associated with hydration, skin barrier function, differentiation and lipid metabolism [49].

One key result showed that IDC upregulated AQP3 mRNA and protein expression [49]. As a member of the aquaporin transmembrane channel proteins expressed in plasma membranes, AQP3 is the predominate aquaporin present in epidermal cells and facilitates movement of water and glycerol between and into cells [55]. Its important role in hydration was demonstrated in AQP3 knockout mice, which displayed lower SC hydration and epidermal glycerol levels, delayed barrier recovery and reduced elasticity compared with wild-type controls [56]. Interestingly, however, while mice studies have shown that topical or systemic application of glycerol could correct these abnormalities [57], at least one clinical study on dry skin showed that application of a vehicle containing an ingredient (glyceryl glucoside) proven to upregulate AQP3 in combination with 6.5% glycerol significantly strengthened skin’s BF beyond that provided by 6.5% glycerol alone in the same vehicle, while hydration improvements were similar [58]. In comparison, topical application of IDC in the absence of glycerol strengthened BF in dry skin and in combination with lower levels of glycerol (2%) improved and prolonged skin hydration more effectively than glycerol alone [49]. It is also notable that while previous studies have isolated AQP3 to basal and spinous layers [55], more recent research has reported the presence of AQP3 as well as AQP10 (another aquaglyceroporin) in human SC, which may have further yet unknown ramifications for SC hydration [59].

IDC also significantly upregulated CD44, a prominent transmembrane glycoprotein that is the principal cell-surface receptor for hyaluronic acid [60]. Hyaluronic acid (HA) is a glycosaminoglycan with exceptional capacity to bind and retain water molecules [61]. While HA has been primarily associated with the extracellular matrix (ECM) of the dermis, HA is also located in the ECM between upper spinous and granular cells of the epidermis and appears to co-localize with CD44 [62]. It is postulated that epidermal HA within the inter-keratinocyte spaces draws water from the dermis to the epidermis to help regulate water balance in skin and help maintain hydration within the SC [61]. Consistent with this is the observation that epidermal but not dermal HA disappears in senescent skin and is considered one of the hallmark histochemical changes that helps account for the loss of moisture in aged skin [61]. Moreover, studies report that abrogation of CD44 expression in keratinocytes in skin of mice results in failure of the epidermis to accumulate HA, with loss of resulting CD44-HA interactions associated with impaired proliferation and differentiation, delayed restoration of BF following acute perturbations and disrupted keratinocyte homeostasis of HA, which resulted in HA accumulation in the superficial dermis with concomitant loss of skin elasticity [63,64,65].

In addition to *AQP3* and *CD44*, IDC also significantly upregulates E-cadherin (*CDH1*), a key component of adherens junctions that promotes strong cell–cell adhesion between keratinocytes and that plays a crucial role in proper assembly of tight junctions (TJs) within the second layer of stratum granulosum [66,67]. As intercellular junctions, TJs function as physical barriers to restrict paracellular passage of water and other pathogens and larger molecules through the skin [66,67]. Indeed, mice lacking E-cadherin in the epidermis die shortly after birth because of dehydration [67].

Another prominent gene found to be stimulated by IDC includes ceramide synthase 3 (*CERS 3*). The enzyme CerS3 plays a pivotal role in regulating composition of epidermal ceramides, which as a class constitute about 50% of epidermal lipids [68]. CerS3 possesses unique ability to synthesize ceramides with ultra-long chain (C26 and longer) fatty acids that are integral in forming lipid lamellae with proper capacity to restrict water loss and prevent penetration of pathogens, allergens and irritants [69]. Deficiencies or dysregulation of CerS3 have been linked to severe skin disorders, including congenital ichthyosis (characterized by impaired barrier function and excessive dryness), whereas functional loss of CerS3 causes lethal skin barrier disruption [70]. Through its control of ultra-long chain ceramides, CerS3 thus operates as a critical regulator of skin health, further highlighting its potential as a therapeutic target in treatment of BF-related skin diseases.

IDC was also found to stimulate genes [49] that encode precursor proteins that play critical roles in formation of the cornified cell envelope, including late cornified envelope proteins 1E and 3D [71] as well as SPRR3 [72], a member of the proline-rich family of proteins (SPRRs). These proteins crosslink with numerous other proteins (loricrin, filaggrin, involucrin) through disulfide and N^ε^-(γ-glutamyl)lysine isopeptide bonds mediated by transglutaminases to form the highly insoluble, tough and resilient matrix that comprises the CE as an integral component of the barrier function. SPRRs constitute about 8% of corneocyte cell proteins [73] and have been identified as key modulators of CE’s strength, toughness and flexibility, which interestingly from separate biophysical measurements have been especially ascribed to SPRR3 compared to other classes of SPRR proteins [74]. Beyond their established effects in cornification, the family of SPRRs are also associated with significant roles in wound healing and tissue repair as antioxidants to regulate excess formation of reactive oxygen species (ROS) [75] and more recently were found to possess antimicrobial defenses [76], among other biological functions [77].

Taken together, it is apparent that IDC through its ability to modulate diverse sets of genes can elicit improvements in keratinocyte differentiation and hydration that favorably impact formation and maintenance of SC barrier function that go beyond physical modes of action typically associated with humectancy or occlusivity of individual molecules.

#### 3.2.3. IDL Represses Expression of Inflammatory Genes and Supplements IDC in Stimulation of Pro-Differentiation Genes to Restore Epidermal Barrier Function and Improve Skin Hydration

While IDC increased the expression of some keratinocyte (KC) differentiation genes and proteins (notably AQP3, CD44, CDH1and precursors of skin cornification proteins), IDL upregulated expression of other genes unaffected by IDC [78]. For example, IDL but not IDC upregulated expressions of *FLG* and *INV* mRNA and increased their protein abundance. Additionally, in a separate ex vivo study, topical application of 4% IDL increased epidermal protein levels not only of AQP9 and FLG by 94% and 13%, respectively, but also of loricrin (LOR) by 12%. FLG is implicated in several aspects relevant for skin barrier function, keratinocyte differentiation and epidermal homeostasis and ultimately gives rise to NMF that provides intracellular humectancy for water retention within corneocytes [79]. Increased production of involucrin early in terminal differentiation plays a critical role in establishing the cornified envelope (CE) and setting up the scaffolding that facilitates the assembly of lamellar lipids in the intercorneocyte spaces. In human skin, LOR constitutes about 65–70% of the protein content of the CE [73], which through crosslinks of disulfide and N^ε^-(γ-glutamyl)lysine isopeptide bonds with other proteins (IVL, SPRRs, FLG, plakoglobin, plakophilin) creates the robust, insoluble matrix of the CE that fosters resilience against water loss, penetration of pathogens or irritants and mechanical stress (shearing, stretching, compressing). Inborn errors in this cross-linking process can impair barrier function and lead to pathological conditions, such atopic dermatitis or ichthyosis [80].

Another important differentiating feature of IDL not found with IDC is its anti-inflammatory activity. IDL was shown to repress inflammatory gene expression [78] associated with T-cell activation, unhealthy skin signature genes (*DUSP7*, *AKR1B10*, *ARNTL2*) and coexpressed IFN response genes within the coexpression module STAT1-57 (*IFI1*, *IFI3*, *ISG15*, *IFI44*, *IFI35*), which become activated in psoriasis and some skin cancers. Interestingly, as previous research also established that corticosteroids repress STAT1-57 genes [81], it was speculated that IDL could likely function through a dual mechanism of action that combines corticosteroids-like anti-inflammatory effects with positive promotion of KC differentiation to restore and maintain skin’s barrier function [78]. With age, the skin’s ability to maintain an effective barrier function declines, making it more susceptible to external stressors. This impaired barrier contributes to chronic low-grade inflammation, a process known as inflammaging. Managing subclinical inflammation is therefore beneficial not only in slowing age-related skin changes associated with inflammaging but also in preventing the progressive buildup of inflammatory signals that may precipitate clinical flares in inflammatory skin disorders such as atopic dermatitis (AD), which is characterized by lifelong cycles of remission and relapse [20,25].

In addition to these effects, other salient findings revealed that IDL increased the expression of genes known to signal early differentiation (*KRT1, KRT10*) and to drive and orchestrate KC differentiation and epithelial morphogenesis (*GHRL1, ZNF750, DSG1*) from the proliferative basal state through to the terminally differentiated state of cornified epithelium. Moreover, IDL increased gene expression not only of *SPRR3* but also of *SPRR4*, both of which are reported to be induced in epithelial tissues upon exposure to environmental stressors, including UV radiation [82] or PM 2.5 small particulate matter [83], and are believed to represent a form of adaptation to bolster the epithelial barrier against further damage in an effort to help preserve its integrity [82].

Consistent with the foregoing results, in a short-term (1–2 weeks) placebo-controlled double-blinded clinical study, topical application of 2% IDL to dry skin was found to reduce TEWL and enhance hydration significantly [78]. In this regard, IDL functions in a complimentary fashion to IDC in its ability to improve skin hydration and strengthen barrier function by virtue of its ability to stimulate a series of pro-differentiation genes.

As mentioned previously, metabolism of IDL in KCs can lead to release of oleic and linoleic acids as free fatty acids. This stems from the fact IDL is manufactured by esterifying the fatty acids present in sunflower seed oil, which comprises high linoleic acid (60–70%) but low oleic acid (20–30%) content, with isosorbide [78]. IDL is 99% pure in diester content comprising a mixture of di-linoleate, di-oleate and linoleate/oleate diesters. While oleic acid exerts disruptive effects on the permeability barrier [84], linoleic acid as an essential fatty acid exhibits many positive benefits associated with enhanced barrier recovery, wound healing and anti-inflammatory effects, as well as an important constituent in therapeutic treatments for hyperpigmentation, acne, xerosis and mild-to-moderate forms of psoriasis and AD [85]. Plus, linoleic acid through esterification to omega-hydroxy fatty acids forms an important subgroup of ceramides with very long chain fatty acids, which are formed solely in the epidermis and play an essential role in structuring intercellular lipids into membrane sheets that are crucial for integrity of the permeability barrier [86]. Notably, failure to synthesize this subgroup of ceramides in essential fatty acid deficiency leads to abnormalities in structure of extracellular lipid lamellae with resulting impairment in barrier function [86].

While linoleic acid possesses multiple skin benefits, gene expression profiling comparing IDL with ethyl linoleate (EL) has established that IDL is not simply a carrier for linoleic acid and that important differences between IDL and El likely arise from formation of isosorbide 5-monoester metabolites from IDL [78]. In general, results substantiated that IDL was more effective than EL at increasing expression of genes associated with KC differentiation, lipid homeostasis and epidermal barrier function. For example, genes encoding key marker proteins (*PCNA*, *KRT1*, *KRT14*, *FLG*) expressed in mitotic and basal layers or during early and late KC differentiation were uniformly but not significantly decreased by EL, whereas IDL increased early and late KC differentiation genes, such as *KRT1* and *SPINK5*. Moreover, IDL was more effective than EL at repressing expression of inflammatory genes, including genes involved with T-cell activation, unhealthy skin signature genes and co-expressed IFN response genes within the STAT1-57 module that becomes activated in psoriasis and some skin cancers.

Collectively, these findings illustrate how IDC and IDL initiate signaling changes in KCs through complementary mechanisms. IDC primarily enhances lipid organization and stimulates early differentiation markers, while IDL engages both pro-differentiation and anti-inflammatory gene networks, including repression of IFN-responsive signatures and STAT1-57–associated modules. By improving the lipid milieu and reducing inflammatory stress, both diesters shift KC signaling toward a barrier-restorative, homeostatic state. This coordinated modulation of differentiation, lipid synthesis, and inflammatory pathways explains how IDC and IDL act as upstream initiators of the transcriptomic and functional improvements observed in AD models and clinical studies.

**Figure 2:**
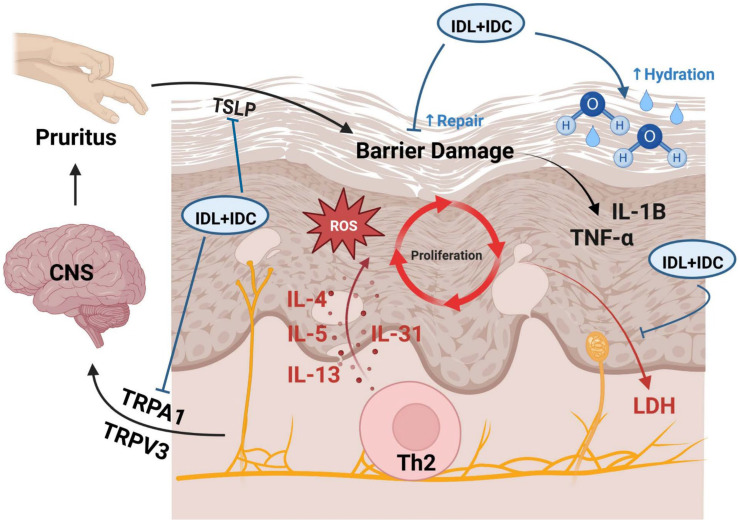
Hypothesized mechanisms by which IDL + IDC improve AD skin lesions. Taken from *Int. J. Mol. Sci.* **2022**, *23*, 14307; https://doi.org/10.3390/ijms232214307 [89]. Th2-driven cytokines (IL-4, IL-5, IL-13, IL-31) and epithelial-derived TSLP disrupt the skin barrier, increase ROS, accelerate keratinocyte turnover, and activate TRPA1-mediated pruritic signaling. IDL + IDC supply fatty acids to strengthen the stratum corneum, enhance hydration, reduce inflammatory cytokine accumulation, down-regulate TRPA1/TRPV3, and normalize epidermal turnover, decreasing LDH leakage into the circulation.

In summary, both IDC and IDL function to improve skin hydration by mechanisms other than passive humectant or occlusive effects. Both IDC and IDL are small lipophilic molecules that function through an inside-out type of mechanism by stimulating diverse series of genes and proteins associated with epidermal hydration, differentiation, lipid synthesis, control of inflammation and formation plus maintenance of barrier function. This renders IDC and IDL promising therapeutic candidates for incorporation into topical formulations, either individually or in combination, for the management of skin conditions characterized by barrier dysfunction, including seasonal xerosis, chronologically aged or photoaged skin, and chronic disorders such as AD. Hypothesized mechanisms by which IDL + IDC improve AD skin lesions is summarized in Figure 2.

### 3.3. IDC + IDL in Management of AD

The pre-clinical and clinical evidence demonstrating the individual effectiveness of IDC and IDL, together with their complementary stimulation of diverse gene sets, provides compelling support for their combined incorporation as specialized ingredients in topical moisturizers aimed at the daily management of AD symptoms. AD is a chronic, relapsing inflammatory skin disease characterized by continuous cycles of remission and eruption of erythematous lesions accompanied by intense itch [13]. AD typically initiates in childhood and frequently resolves during adolescence. If it persists in adulthood, AD can become a lifelong disease that requires vigilant care and management [13,31]. While not completely elucidated, research captures that the pathogenesis of AD is complex, involving intricate interplay between genetic, immunological and environmental factors wherein epidermal barrier dysfunction has been proposed to play a prominent role in development of the disease [15,20,22]. Defects in the BF lead to elevated SC pH, increased TEWL, reduced hydration, alterations in the skin microbiome, and greater susceptibility to environmental irritants and allergens, all of which drive inflammation. In AD, this inflammation shifts the immune response toward a Th2-dominant rather than a Th1-dominant state [15,20,22]. In turn, the resulting inflammation disrupts terminal differentiation, thereby perpetuating barrier dysfunction in a self-reinforcing cycle known as the “outside-to-inside-back-to-outside” hypothesis [21].

Treatment guidelines for AD depend on disease severity but generally include targeted use of anti-inflammatory drugs to calm inflammation and pruritus in conjunction with adjuvant application of nonprescription moisturizers to help restore, maintain and even strengthen skin’s BF to reduce incidence of and prolong periods between disease flare-ups [20,31]. Multiple clinical studies document the efficacy of purposefully designed moisturizers to prevent relapses, increase time-to-relapses, reduce the number of flared lesions and significantly improve quality of life scores [37,87]. However, studies also document that all moisturizers are not created equal in their ability to impart these benefits to AD skin [35,36,37,38,39,40]. Care must be exercised to differentiate between moisturizers that ameliorate dry skin symptoms in normal skin from those capable of delivering more therapeutic effects required for AD treatments. Toward this objective, specific functional mechanistic studies have been described that characterize impact of moisturizers on critical barrier function properties (hydration, TEWL, skin surface pH, SC integrity and cohesion) following daily applications for 1 month on AD skin of mild to moderate severity. These mechanistic studies provide important methodology that can discern effectiveness between moisturizers and whether their daily application (1) further impairs BF (which helps perpetuate barrier dysfunction), (2) transiently improves BF during application (ca. 6 h) but otherwise does not damage BF or (3) stimulates BF repair and strengthening to sustain BF properties over protracted durations [39,40]. In AD skin, use of effective moisturizers is even more important since research documents that even areas of non-lesional, normal-looking skin still exhibit diminished BF properties, subclinical inflammation [22] and elevated mitochondrial activity with increased oxidative stress [88].

In tissue culture models of AD-like skin, established by treating keratinocytes with a combination of Th2 cytokines (IL-4, IL-13, IL-31) and TNF-α, co-treatment with IDC and IDL was shown to counteract multiple cytokine-induced features characteristic of atopic dermatitis [89]. Specifically, topical application of IDC + IDL preserved epidermal barrier function, mitigated dysregulation of keratinocyte (KC) differentiation by upregulating early (but not late) KC differentiation markers and suppressed TNF-α–responsive genes. In addition, the combination synergistically downregulated key pro-inflammatory (IL1B, ITGA5) and neurogenic pruritus (TRPA1) mediators and significantly reduced the secretion of lactate dehydrogenase (LDH), a marker of cellular damage. Collectively, these findings suggest that IDC + IDL treatment promotes tissue integrity, reduces inflammation, and alleviates pruritic signaling, indicating a favorable therapeutic response for AD-like conditions.

In clinical investigations, the combination of IDC and IDL in topical formulations was evaluated in two single-center, randomized, double-blind, vehicle-controlled trials involving patients with mild-to-moderate AD: one trial conducted in adults over a 4-week period [90] with one-week regression and the other trial in children over an 8-week period [91]. In each study, subjects were allowed to use topical corticosteroids (TCS) as needed to manage disease flares and were instructed to apply either a moisturizer without IDC + IDL as vehicle control or the moisturizer with IDC + IDL to the entire body once daily over the course of the study. In the adult trial, results highlighted that after 4 weeks the moisturizer with IDC + IDL compared with its vehicle control significantly improved itch (65.6% vs. 43.8%, *p* = 0.013) and EASI 75 (75% improvement in the Eczema Area and Severity Index score) (56.5% vs. 25%, *p* = 0.07) as primary outcomes [89]. Additionally, over the course of the 4-week study, use of the IDC + IDL moisturizer compared with the vehicle control led to significant reductions in relative use of TCS, which manifested after week 1 (24.8% vs. 292.5%, *p* = 0.039), peaked at week 2 (46% vs. 220%, *p* = 0.08) and then persisted through week 4, and in relative abundance of *S. aureus* (*p* = 0.044). Notably, significant improvements in sleeplessness ratings in a sub-set of subjects who suffered moderate-to-severe sleep disturbances (52.8% vs. 25.9%, *p* = 0.02) were also recorded. As both moisturizers contained 2% glycerin and 0.3% lactic acid as effective humectants, both placebo and IDC + IDL vehicles significantly increased skin hydration relative to baseline but were not significantly different from each other although the IDC + IDL lotion was directionally greater. Interestingly, after 4 weeks, while TEWL values were not statistically different, the values relative to baseline for vehicle control increased whereas those for IDC + IDL decreased in a manner consistent with improvements in barrier function.

Consistent with findings in adults, application of the IDC + IDL moisturizer in children aged 2 to 17 years resulted in significantly greater improvements in atopic dermatitis management compared to the vehicle control [91]. In particular, after 8 weeks, these included significant improvements in proportion of subjects who achieved EASI 50 scores (81.0% vs. 53.9%, *p* = 0.10) and 4-point reductions in subjective itch (42.9% vs. 12.5%, *p* = 0.045), in reducing dependence on TCS throughout the study (3.4 g vs. 13.3 g, *p* = 0.012), in increasing values of skin hydration relative to baseline in the IDC + IDL group (68.3%, *p* = 0.0029) but not in the control group (35.4%, *p* = 0.181)) and in reducing relative abundance of *S. aureus* in contrast to the control group which elicited no changes. These improvements also led to a reduction (−1.24, *p* = 0.035) in values for DLQI (Dermatology Life Quality Index) after 8 weeks in the IDC + IDL group that was not observed in the control group.

Two of the attributes influenced by IDC + IDL from these clinical trials in adult and pediatric AD populations merit further comment. One attribute is the steroid-sparing benefit of IDC + IDL [90,91], which aligns with its anti-inflammatory properties observed in previous studies using tissue culture models of AD [89] where co-treatment of IDC + IDL downregulated inflammatory mediators (IL1B, ITGA5) as well as neurogenic pruritic mediators (TRP1, TRPV3, TSLP). These actions position IDC + IDL as botanically derived alternatives for inclusion in moisturizers to help manage sub-clinical inflammation and pruritus before their symptoms manifest on skin, thus avoiding the need to use TCS. This has special relevance to sufferers and caregivers alike who have expressed grave concerns associated with chronic intermittent use of TCS, owing to their association with initiation of barrier damage and skin atrophy, burning or stinging, steroid rebound with flare-ups after cessation of use as well as risk of systemic absorption and adrenal suppression [92].

The second attribute is the ability of IDC + IDL to reduce the abundance of *S. aureus* and help normalize skin’s microbiome following 4- or 8-week application [90,91]. Recurrent *S. aureus* colonization is a hallmark of AD and is recognized today as one of the most influential environmental factors in development of the disease [93]. Through multiple pathways, *S. aureus* in AD triggers acute flares and exacerbates skin condition further by broadly disrupting an already weakened barrier function, amplifying Th-2/T17 driven inflammation and reducing richness and diversity of the microbiome at the expense of beneficial bacterial strains, such as *S. epidermidis* [93]. Research also shows a strong correlation between *S. aureus* density and disease severity in AD, with higher loads linked with more severe symptoms [94]. As skin colonization by *S. aureus* starts with adhesion to the skin, which is favored by skin dryness, increased TEWL and elevated values of skin surface pH (i.e., a damaged barrier function), it is not surprising that daily application of moisturizers are recognized today as an important component of a long-term strategy in clinical management of AD to improve patient outcomes [95]. Several studies captured in a recent review support the efficacy of daily moisturizers to reduce *S. aureus* abundance and generally ascribe their efficacy to barrier restoration features (increasing hydration, decreasing TEWL, decreasing skin surface pH, repairing lipid bilayers), which impede the ability of *S. aureus* to adhere to the skin [95]. In this regard, it is notable that compared to its vehicle control, only the lotion with IDC + IDL elicited beneficial shifts in skin microbiome, which may indicate that IDC + IDL promotes a stronger, more resilient barrier function [90,91].

In summary, the significant improvements observed in EASI scores, itch reduction, decreased reliance on corticosteroids, reduced abundance of *S. aureus*, and improved sleep quality can ultimately be attributed to the presence of IDC and IDL in the moisturizing lotion (Table 1) and their combined ability to modulate gene pathways involved in repairing, strengthening, and hydrating the skin, thereby supporting both barrier integrity and moisture retention [90,91].

#### 3.3.1. Therapeutic Outlook of IDC + IDL in Inflammatory Skin Disorders

Isosorbide-based fatty acid diesters, particularly IDC and IDL, offer a novel dual-action therapeutic approach for inflammatory skin diseases such as AD. Preclinical investigations using cytokine-induced keratinocyte models of AD have shown that IDC and IDL exert synergistic effects that preserve epidermal barrier integrity, mitigate dysregulated keratinocyte differentiation, and suppress key inflammatory mediators, including IL1B, ITGA5, TSLP, TRPV3 and TRPA1. Additionally, reductions in lactate dehydrogenase (LDH) secretion suggest decreased cellular stress and damage, further underscoring their tissue-protective potential. LDH inhibitory effect is particularly important in the subset of patients with high serum LDH, who appear less likely to show long-term improvement with certain biologics such as Dupixent.

Importantly, IDC and IDL engage distinct signaling pathways to enhance barrier function while dampening pro-inflammatory and pruritic responses. This is particularly significant given the complex pathogenesis of AD, which involves epidermal barrier dysfunction, Th2-driven inflammation, and microbial dysbiosis. Through normalization of stratum corneum cohesion, TEWL, and skin surface pH, IDC and IDL modulate both the “outside-in” and “inside-out” components of disease. Their capability to provide sustained barrier support alongside inflammation attenuation highlights a favorable therapeutic profile, making them strong candidates for incorporation into long-term, non-steroidal topical maintenance regimens.

#### 3.3.2. Clinical Implications and Future Directions for IDC + IDL in Dermatology

Clinical evidence strongly supports incorporating IDC and IDL into topical moisturizers as effective adjunctive therapies for mild-to-moderate atopic dermatitis (AD) across both pediatric and adult populations. Two randomized, double-blind, vehicle-controlled clinical trials demonstrated significant improvements in itch severity, EASI scores, and Dermatology Life Quality Index (DLQI), along with a reduced reliance on TCS. Notably, IDC + IDL formulations also produced a statistically significant reduction in *Staphylococcus aureus* colonization, a key factor driving AD pathophysiology and flare severity. Mechanistically, IDC + IDL upregulates the expression of critical barrier proteins, including FLG, IVL, and LOR, as confirmed by both differential gene expression (DEG) analyses and protein-level validation. These findings reinforce the role of IDC + IDL in restoring barrier integrity at a molecular level, complementing their demonstrated clinical efficacy.

Two clinical advantages warrant particular emphasis. First, the steroid-sparing effect of IDC + IDL directly addresses concerns over long-term TCS use, including barrier disruption, cutaneous atrophy, and systemic side effects. By targeting subclinical inflammation and neurogenic pruritus, IDC + IDL formulations offer a proactive approach to mitigating symptom recurrence. Second, their ability to restore barrier function and normalize the skin microbiome aligns with emerging paradigms linking microbial balance to chronic inflammatory skin diseases.

#### 3.3.3. Limitations and Future Clinical Studies

While current evidence supports the therapeutic potential of isosorbide fatty acid diesters, particularly IDC and IDL, several important limitations must be acknowledged. To date, only two clinical studies have evaluated IDC + IDL formulations in AD. Both were single-site trials with small sample sizes, which restricts statistical power and limits the generalizability of the findings across diverse populations and disease severities. Larger, multicenter studies are needed to validate these early outcomes and strengthen the clinical evidence base. Additionally, many of the mechanistic insights presented such as the modulation of inflammatory mediators, barrier-related genes, and pruritic pathways are derived primarily from gene-expression studies using in vitro keratinocyte models of atopic dermatitis. While these models provide valuable translational insights, confirmatory studies in human skin and clinical settings are essential to fully substantiate these mechanistic pathways.

Future clinical investigations should therefore prioritize large-scale, multicenter randomized controlled trials to evaluate the efficacy, safety, and durability of IDC + IDL across a broader demographic spectrum, including pediatric, adult, and ethnically diverse populations. Longitudinal trials assessing long-term barrier restoration, flare prevention, and steroid-sparing durability will be especially important in understanding their role in chronic disease management. Comparative effectiveness studies against established non-steroidal topical agents may further contextualize the therapeutic placement of IDC + IDL within current AD treatment algorithms.

Mechanistic clinical studies that further integrate non-invasive biomarker assays, and lesional transcriptomic analyses will help elucidate the pathways through which IDC + IDL exert their synergistic barrier-restorative and anti-inflammatory effects in vivo. Furthermore, evaluating their potential in other barrier-deficient dermatoses, including psoriasis, seborrheic dermatitis, chronic hand eczema, and inherited barrier disorders, may reveal broader dermatologic applications.

Overall, while preliminary findings are promising, more robust and comprehensive clinical research is necessary to confirm the therapeutic value of IDC + IDL and to define their optimal use across inflammatory and barrier-compromised skin diseases.

### 3.4. IDMF in Management of Psoriatic Skin

Treatment options for psoriasis have significantly expanded in recent decades as disease mechanisms have become better understood. However, most progress has been made in developing systemic agents, particularly biologics, while advancements in topical therapies remain limited. To address this limitation, a novel isosorbide derivative, IDMF, was designed recently to retain DMF’s anti-psoriatic efficacy while eliminating its skin-sensitizing properties [96].

Preclinical findings indicated that IDMF lacked discernible genotoxicity and phototoxicity in cultured skin cells and exhibited minimal to no sensitization in rat, rabbit, and guinea pig models. Notably, IDMF significantly reduced erythema and scaling in psoriasiform lesions induced by topical IMQ in mice, suggesting both tolerability and anti-psoriatic efficacy. These effects may be mediated through repression of cytokine responses (IL-17A and TNF), inhibition of proinflammatory transcription factors (NF-κB) and activation of NRF2/ARE pathways. These findings supported early-phase clinical studies to evaluate IDMF as a topical anti-psoriasis therapy [96].

Additionally, IDMF demonstrates markedly lower chemical reactivity than DMF, primarily due to steric hindrance imposed by its rigid, bicyclic isosorbide backbone. This steric bulk limits the easy accessibility of the fumarate ester moieties to nucleophilic attack by endogenous biomolecules such as thiol or amine groups on skin proteins. Such nucleophilic interactions are a critical initiating event in the molecular pathway leading to skin sensitization. The reduced reactivity of IDMF is further reflected in its higher activation energy for electrophilic reactions, indicating a greater energetic barrier for covalent bond formation with biological nucleophiles. Consequently, IDMF does not exhibit the sensitizing properties associated with DMF, making it a safer alternative for topical applications where skin tolerance is a key requirement [96].

Since DNA repair interference has been proposed as the principal mechanism of DMF-induced contact dermatitis [97], research efforts investigated the effects of IDMF on cyclobutane pyrimidine dimer (CPD) formation [96]. CPDs represent the main class of DNA lesions that correlate strongly with induction of melanoma and non-melanoma skin cancers and arguably characterize the most important type of photodamage caused by sun exposure [98,99,100]. CPDs are highly genotoxic lesions linked to skin cancer and cellular senescence [101,102]. Interestingly, DMF significantly reduced CPD repair, whereas CPD formation in IDMF-treated cells did not differ significantly from controls. Moreover, IDMF did not provoke inflammation in normal tail skin, which likely explains the absence of sensitization in animal models [96].

DMF exhibits a stronger direct antiproliferative effect on KCs, potentially due to its unique metabolites, such as methanol [103,104], whereas other research observed a direct anti-inflammatory effect of IDMF on cytokine-stimulated KCs, evidenced by repression of key cytokine pathways, including TNF-α, IL-17A, IL-23A, IL-36G, and IFN-γ [96]. These effects resemble those previously described for dimethyl fumarate (DMF) and monomethyl fumarate (MMF). For instance, MMF directly applied to TPA-activated mouse keratinocytes (KCs) suppressed TNFα, IL6, and IL1a mRNA expression, while in human KCs, MMF reduced TNF-α protein below detection limits [105]. Similarly, DMF treatment of IFN-γ or PMA-stimulated KCs downregulated CXCL8, CXCL9, and CXCL10 [106]. Thus, IDMF’s direct anti-inflammatory action may disrupt local inflammatory circuits, thus preventing immune cell recruitment into psoriatic lesions.

NF-κB inhibition is proposed as an NRF2-independent mechanism underlying DMF’s anti-psoriatic effects [107]. DMF has been shown to inhibit NF-κB in various cell types, including T cells [108]), dermal fibroblasts [109], and KCs [110]. DMF, however, is a stronger NF-κB inhibitor than its metabolites, meaning any direct NF-κB modulation in KCs would not occur when DMF is administered orally [111]. Previous studies also indicated that DMF can inhibit AP-1 in lung fibroblasts [112]. Furthermore, motif analysis identified NF-κB and AP-1 binding sites in genes downregulated by IDMF, including IL23A, which suggests that IDMF like DMF may repress NF-κB activity. However, with topical application, this repression occurs locally, reducing IL23A and other NF-κB target genes. IDMF may also interact with other transcription factors, such as AP-1, STAT, or IRF1, identified in motif analysis [96].

Oxidative stress plays a key role in psoriasis lesion development [113]. Neutrophils contribute to this accumulation by undergoing respiratory bursts that release superoxide anion [114]. Depending on its environment and reaction partners, superoxide anion can act as an oxidant, a reductant, a nucleophile, or an enzyme substrate. NRF2 constitutes a central regulator of cellular defense mechanisms, including the regulation of antioxidant, anti-inflammatory, and mitochondrial pathways, making it a highly attractive therapeutic target of disease modification. Indeed, activation of the NRF2-ARE pathway is considered an important anti-psoriatic mechanism of DMF [115]. DMF and MMF activate NRF2 in KCs [116] and represses NRF2 targets in psoriasis lesions [117]. Interestingly, activation of Nrf2/Keap1 pathway by oral DMF administration has been reported to alleviate oxidative stress and age-associated infertility in mice [118]. Based on luciferase gene assays, IDMF was reported to have a stronger NRF2 activation than DMF, upregulating oxidative stress response and GSH metabolism genes. IDMF may therefore share antioxidant mechanisms with DMF/MMF, but via topical application, it may act directly on ROS produced by epidermal neutrophils [119].

The IMQ-induced psoriasiform mouse model is widely utilized for evaluating anti-psoriatic compounds and investigating disease mechanisms [120]. In this model, IDMF significantly alleviated erythema, scaling, and epidermal thickening in IMQ-induced lesions while suppressing genes associated with immune activation. While DMF is a well-known skin sensitizer [121], IDMF does not exhibit this property.

Collectively, these findings clarify how IDMF initiates signaling changes in psoriatic skin through coordinated modulation of redox balance and inflammatory transcriptional networks. By activating NRF2, IDMF enhances antioxidant defense programs that reduce oxidative stress and weaken upstream triggers of cytokine-driven inflammation. In parallel, IDMF suppresses NF-κB, AP-1, IRF1, and STAT-linked pathways, thereby downregulating IL-17A, IL-23A, IL-36G, IFN-γ, and other psoriasis-associated cytokines at their transcriptional origins. This dual modulation of oxidative and immune signaling interrupts the pathogenic cycle of keratinocyte hyperproliferation, neutrophil recruitment, and epidermal thickening. Through these redox-driven and immunoregulatory mechanisms, IDMF functions as an upstream initiator of the molecular improvements observed in keratinocyte assays and IMQ-induced psoriasiform lesions.

#### 3.4.1. Therapeutic Outlook of IDMF to Manage Psoriasis

IDMF represents a next-generation topical agent designed to retain the therapeutic benefits of DMF while minimizing its adverse skin-sensitizing effects. The rigid, bicyclic isosorbide scaffold introduces steric hindrance that markedly reduces electrophilic reactivity, resulting in a significantly lower propensity for nucleophilic interactions with skin proteins. This design translates into a higher activation energy for covalent bond formation and underpins IDMF’s improved safety profile.

Preclinical studies have demonstrated that IDMF effectively suppresses hallmark features of psoriasis in the IMQ-induced mouse model, including erythema, scaling, and epidermal thickening. Mechanistically, IDMF modulates both redox and inflammatory signaling pathways. It activates the NRF2/ARE axis more robustly than DMF, enhancing the transcription of antioxidant and glutathione metabolism genes. Simultaneously, IDMF represses pro-inflammatory transcription factors, including NF-κB, AP-1, and potentially IRF1, leading to the downregulation of key psoriasis-associated cytokines such as IL-17A, IL-23A, IL-36G, and IFN-γ.

Unlike systemic DMF, which relies on hepatic metabolism and induces dose-limiting side effects, IDMF is suitable for topical application, offering site-specific activity with reduced systemic exposure. This dual mechanism—potent NRF2 activation coupled with NF-κB suppression—provides a balanced therapeutic approach, addressing both oxidative stress and immune dysregulation within psoriatic lesions.

#### 3.4.2. Clinical Implications and Future Directions of IDMF in Psoriasis Treatment

The pharmacological properties of IDMF position it as a promising topical therapy for psoriasis and potentially other inflammatory dermatoses. Its ability to modulate epidermal inflammatory pathways and restore redox homeostasis locally presents a strategic advantage over existing topical agents, which often lack disease-modifying potential. In contrast to DMF, IDMF does not inhibit DNA repair mechanisms or provoke phototoxicity, sensitization, or inflammation in healthy skin, underscoring its safety profile for chronic topical use.

Given its efficacy in KC and animal models, IDMF may fulfill a critical gap in current therapeutic strategies, particularly for patients with mild-to-moderate plaque psoriasis who are not candidates for systemic biologics. By targeting keratinocyte-driven cytokine signaling and reducing neutrophil-derived oxidative stress, IDMF holds potential not only for symptom control but also for modifying disease trajectory. As our understanding of the molecular underpinnings of psoriasis evolves, IDMF represents a rationally engineered therapeutic that integrates anti-inflammatory and antioxidant functions into a topically deliverable, non-sensitizing platform. Its clinical translation may open new avenues for precision topical therapy in dermatology.

#### 3.4.3. Limitations and Future Clinical Studies

Current evidence for IDMF is limited to preclinical models, including in vitro KC studies and the IMQ-induced mouse model. While these systems provide strong proof-of-concept for IDMF’s antioxidant and anti-inflammatory mechanisms, they cannot fully replicate the molecular and clinical heterogeneity of human psoriasis. Moreover, many mechanistic insights, such as NRF2 activation and suppression of NF-κB, AP-1, and IRF1 are derived primarily from gene expression analyses rather than human clinical tissue studies.

A key limitation is the absence of human clinical data, as no trials have yet evaluated IDMF for safety, tolerability, or efficacy in patients with psoriasis. Important factors -including optimal dosing, long-term local tolerability, percutaneous absorption, and comparative performance against standard topical therapies remain unknown.

Future research should prioritize early-phase clinical trials to establish foundational safety and efficacy data in mild-to-moderate plaque psoriasis. These studies should incorporate translational endpoints, such as non-invasive biomarker assessments and lesional transcriptomic profiling, to validate IDMF’s proposed mechanisms in human skin. Larger, multicenter trials will ultimately be required to define its therapeutic potential and positioning within psoriasis treatment algorithms.

## 4. Systemic Applications of IDMF

### 4.1. Rationale for Systemic Evaluation

Although IDMF was initially designed and evaluated as a topically applied derivative of DMF, its molecular profile suggests potential utility beyond localized skin treatment. The preclinical data presented in the preceding sections demonstrate that IDMF retains key DMF-associated pharmacological activities, most notably NRF2 pathway activation and NF-κB suppression, while reducing electrophilic reactivity and improving tolerability. These mechanisms, together with IDMF’s favorable safety characteristics in topical formulations, provide a strong rationale for exploring whether IDMF could be translated into systemic therapeutic contexts where oxidative stress and immune dysregulation play central roles. Given the broad clinical utility of fumarate-class drugs, particularly DMF, the question naturally arises as to whether IDMF’s improved chemical stability and reduced sensitization potential may offer advantages for systemic use. This consideration provides the foundation for examining the potential systemic applications of IDMF, as outlined in the following section.

### 4.2. Systemic Therapeutic Landscape of Fumarates

DMF is a well-established, dual-indication therapeutic agent for treatment of MS and psoriasis [122]. Recent comprehensive reviews highlight DMF’s expanding therapeutic roles and focuses on its mechanisms of action, particularly activation of the nuclear factor erythroid 2–related factor 2 (Nrf2) and inhibition of NF-κB signaling [123,124]. These pathways underscore DMF’s antioxidative and immunomodulatory properties, which contribute to its therapeutic efficacy in a range of chronic diseases, including cardiovascular and respiratory disorders, cancers, ocular conditions, neurodegenerative diseases, and systemic inflammatory and immune-mediated pathologies. Another review further provides an updated and broader perspective on DMF’s pharmacological applications, building upon the foundational insights of previous research.

**Figure 3:**
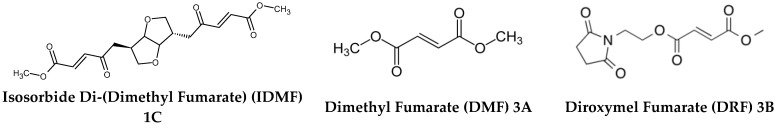
Structures of fumarate-based compounds for treatment of multiple sclerosis.

The clinical success of DMF has stimulated the development of novel fumarate derivatives aimed at preserving its efficacy while improving tolerability. One such advancement is Diroxymel Fumarate DRF (3B), which has demonstrated improved gastrointestinal tolerability compared with DMF in patients with relapsing-remitting MS [125]. Nevertheless, adverse effects such as facial flushing and gastrointestinal symptoms remain common across clinical trials [126,127]. Facial flushing and GI events are common with DRF, typically occurring in about one-third to nearly half of patients, especially in the first month. In addition to its potential as a novel topical delivery platform for targeted treatment in psoriasis, IDMF also represents a structurally distinct compound for other fumarate-based treatments, as shown in Figure 3 by its structural comparison with DMF and DRF.

### 4.3. IDMF Mimics DMF-Induced Astrocyte Transcriptomes While Suppressing Reactive Phenotype-Associated Genes

A recent study compared the transcriptomic effects of IDMF (1C) and DMF (3A) in primary human astrocytes, which revealed that both compounds activated the redox-sensitive Nrf2 pathway. In addition, however, IDMF also downregulated genes associated with a reactive astrocyte phenotype, suggesting a potentially more targeted and beneficial therapeutic profile [128]. This article directly addresses how IDMF not only mimics DMF in astrocyte gene expression but also suppresses reactive phenotype-associated genes. Key highlights include:

- IDMF preserves NRF2-driven antioxidant defenses as a core mechanistic overlap between IDMF and DMF. Through comprehensive global transcriptome profiling, the study demonstrates that IDMF closely mirrors the gene expression signature elicited by DMF in human astrocytes, particularly in pathways related to cytoprotection, oxidative stress mitigation, and redox homeostasis. Central to this shared response is the robust induction of the NRF2 transcriptional program, a critical determinant of cellular defense against oxidative and electrophilic stress [129]. Within cells, NRF2 serves as a crucial transcription factor and plays an essential role in maintaining the balance of oxidative-reductive reactions and responding to oxidative stress.
- Both IDMF and DMF significantly upregulate canonical NRF2 target genes—including HMOX1 (heme oxygenase 1), NQO1 (NAD(P)H quinone dehydrogenase 1), and GCLM (glutamate-cysteine ligase modifier subunit). These genes are instrumental in the detoxification of reactive oxygen species (ROS), the maintenance of glutathione levels, and the reinforcement of antioxidant capacity in glial cells. Their consistent activation underscores that IDMF effectively recapitulates the protective molecular footprint that underlies DMF’s established clinical benefits, particularly in the treatment of neuroinflammatory conditions like multiple sclerosis.
- Importantly, this conserved activation of NRF2 signaling suggests that IDMF retains the core mechanistic axis responsible for promoting neuronal resilience and limiting oxidative damage, making it a viable and mechanistically grounded alternative to DMF. By preserving this antioxidant foundation, IDMF maintains a proven therapeutic modality while offering a platform for further refinement and enhancement of fumarate-based treatments. Besides its well-known antioxidant and cytoprotective role, an Nrf2 activator also enhances protein quality control, helping cells detect, tag, and remove damaged proteins through proteasomal and autophagic pathways [130].
- IDMF provides targeted suppression of neurotoxic astrocyte signatures as a distinct anti-inflammatory advantage over DMF. Crucially, while IDMF shares core NRF2-mediated antioxidant functions with DMF, it also diverges sharply in its regulation of astrocyte inflammatory programs, revealing a more refined and targeted therapeutic profile. Transcriptomic analyses uncovered that IDMF selectively downregulates gene networks associated with the A1 reactive astrocyte phenotype, a pro-inflammatory, neurotoxic state that contributes to neuronal degeneration and disease progression in multiple neurological disorders [131].
- Most notably, IDMF exerts strong repressive effects on key A1 astrocyte markers, including C3 (complement component 3), SERPING1 (serpin family G member 1), and GBP2 (guanylate binding protein 2) [128]. These genes play key roles in the amplification of CNS inflammation, initiation of the complement cascade, and promotion of astrocyte-mediated neurotoxicity. Unlike DMF, which shows minimal modulation of these transcripts, IDMF demonstrates a precise anti-inflammatory action, actively attenuating the molecular signature of pathological astrocyte activation.
- This selective repression of reactive astrocyte markers positions IDMF as a fumarate compound with enhanced neuroprotective capacity—not only mitigating oxidative damage but also interrupting key inflammatory feedback loops within the CNS glial environment. The data suggests that IDMF can more effectively blunt the progression of neuroinflammation and limit collateral damage to neurons and oligodendrocytes, offering potential advantages in the treatment of conditions characterized by chronic glial activation, such as multiple sclerosis, Alzheimer’s disease, and traumatic CNS injury. By modulating both redox balance and inflammatory gene networks with greater precision, IDMF represents a next-generation therapeutic agent capable of delivering dual-action protection: antioxidant reinforcement and active suppression of astrocyte-mediated neuroinflammatory cascades.

Thus, while maintaining the hallmark antioxidant properties of DMF through robust NRF2 pathway activation, IDMF also introduces a distinct and therapeutically advantageous layer of immune modulation. Unlike DMF, which primarily acts through oxidative stress mitigation, IDMF exhibits a more selective and potent repression of pro-inflammatory gene expression, especially in pathways associated with astrocyte reactivity and neuroimmune signaling [128].

This dual-action profile of cytoprotective activation combined with targeted suppression of neuroinflammatory cascades—establishes IDMF as a mechanistically differentiated compound within the fumarate products. Transcriptomic analyses indicate that IDMF not only mitigates oxidative stress but also actively suppresses astrocyte—mediated inflammatory responses—pathological processes increasingly recognized as central drivers of neurodegeneration in disorders such as multiple sclerosis, Alzheimer’s disease, and Parkinson’s disease. Moreover, by avoiding broad immunosuppression and instead focusing on pathogenic gene networks specific to reactive astrocytes, IDMF presents a refined therapeutic strategy. This selectivity could translate to a lower incidence of systemic side effects, such as lymphopenia or gastrointestinal intolerance, common drawbacks associated with DMF therapy [132].

Collectively, the findings presented here position IDMF as a next-generation fumarate with an optimized molecular profile: one that couples established efficacy in redox regulation with enhanced, cell-type–specific immunomodulatory precision. This mechanistic sophistication holds promise for greater therapeutic efficacy, improved tolerability, and broader applicability across a range of neuroinflammatory and neurodegenerative conditions [128].

### 4.4. Transcriptomic Profiling of Astrocytes Reveals Distinct Responses to IDMF over Traditional Fumarates

Another study [133] presented a comprehensive transcriptomic analysis of fumarate compounds including DRF, MMF, and IDMF, on cultured astrocytes, with a primary focus on IDMF as a novel fumaric acid ester. Using human keratinocytes, the researchers compared IDMF’s gene expression profile against the other two fumarates, which highlighted its potential as a therapeutic agent in multiple sclerosis. Key observations included:

- IDMF demonstrated distinct and potent regulatory effects on a suite of genes associated with the NRF2, NF-κB, and IRF1 signaling pathways comprising key molecular hubs that govern cellular responses to oxidative stress, inflammation, and immune activation. These pathways are critically involved in the pathogenesis of numerous inflammatory and autoimmune skin diseases. Through transcriptomic profiling, the study revealed that IDMF induces a unique expression pattern, markedly different from the other fumarate compounds.

Most notably, IDMF elicited a strong and selective upregulation of NRF2-responsive genes, including those involved in antioxidant defense, detoxification, and maintenance of cellular redox homeostasis (e.g., HMOX1, NQO1, GCLM). This suggests a superior activation of the NRF2 pathway relative to DMF, pointing to a heightened ability to neutralize oxidative stress and protect against cellular damage.

In parallel, IDMF exerted a profound suppressive effect on pro-inflammatory genes regulated by the NF-κB and IRF1 pathways—two central mediators of innate immune responses, cytokine production, and inflammatory amplification. Specifically, IDMF downregulated key cytokines and interferon-stimulated genes (e.g., IL1B, CXCL10, IFIT1) more effectively than DMF, highlighting its broad anti-inflammatory and immunomodulatory capacity.

This dual action of potent NRF2 activation alongside strong repression of NF-κB and IRF1 signaling is particularly significant, as it reflects a balanced therapeutic profile by promoting cytoprotective, antioxidant defenses whilst simultaneously dampening the pathological inflammation central to skin diseases like psoriasis [134]. These findings collectively position IDMF as a compelling candidate for further preclinical and clinical development, offering the potential for enhanced therapeutic efficacy with fewer inflammatory or oxidative side effects compared to existing fumarate-based treatments.

- IDMF’s distinct transcriptomic profile reveals broader and targeted benefits. Gene set enrichment and pathway analyses revealed that IDMF’s transcriptomic signature diverges markedly from that of DMF, reflecting fundamental differences in biological activity at the molecular level. While both compounds modulate pathways linked to oxidative stress and immune regulation, IDMF exhibits a broader and more targeted impact on key gene networks, particularly those associated with anti-inflammatory responses, interferon regulation, and cellular detoxification. Notably, IDMF activates NRF2 target genes more robustly while concurrently exerting greater suppression of pro-inflammatory mediators governed by the NF-κB and IRF1 axes [133].

Thus, IDMF’s mechanistic differentiation promises additional clinical benefits in treatment of inflammatory skin diseases. This divergence suggests that IDMF operates through a distinct and potentially more refined mechanism of action, enabling it to attenuate inflammatory signaling while preserving or even enhancing cytoprotective pathways. As such, IDMF is positioned as a next-generation fumarate compound with substantial therapeutic potential for the treatment of inflammatory disorders, such as multiple sclerosis, psoriasis or atopic dermatitis. Furthermore, its gene expression profile implies a favorable risk-benefit balance, indicating the possibility of improved efficacy and reduced off-target effects or systemic toxicity compared to currently approved fumarates like DMF or DRF [133].

### 4.5. Benchmarking IDMF Against Fumarates in Three Disease-Relevant Models

Recent advances in the development of fumarate derivatives have expanded therapeutic possibilities for chronic inflammatory diseases such as MS and psoriasis. In this context, a recent study [135] further provides a compelling preclinical evaluation of IDMF, exploring its efficacy across three distinct disease-relevant biological models: (1) an oxidative stress model in KCs to mimic psoriatic skin inflammation, (2) a microglial inflammation model relevant to neuroinflammation in MS and (3) a peripheral blood mononuclear cell (PBMC) assay to evaluate systemic immune modulation. Notably, IDMF demonstrated greater potency and a broader activity spectrum than DMF in all three models, with key findings and implications including:

- Oxidative stress model in keratinocytes: IDMF more effectively upregulated antioxidant response elements and suppressed pro-inflammatory cytokines such as IL-6 and IL-8, indicating superior skin-protective properties.
- Inflammation model in the microglial: IDMF significantly inhibited LPS-induced TNF-α and IL-1β production, suggesting enhanced neuroprotective potential relevant for demyelinating CNS disorders.
- PBMC assay: IDMF modulated PBMC cytokine profiles in a manner consistent with an anti-inflammatory phenotype, including suppression of Th1/Th17—associated cytokines, which are crucial mediators in the pathogenesis of both MS and psoriasis.

Moreover, gene expression profiling revealed that IDMF induces unique transcriptomic signatures beyond those triggered by DMF, potentially reflecting engagement of additional signaling pathways. These findings suggest that IDMF may represent a mechanistically distinct and more comprehensive immunomodulator compared to its parent compound.

### 4.6. Mechanistic Basis of IDMF Action in Neuroinflammation and Multiple Sclerosis

IDMF’s mechanistic relevance extends into the central nervous system, where its actions on astrocytic redox and inflammatory pathways have direct implications for MS. IDMF initiates its therapeutic activity in neuroinflammatory settings by modulating core redox-regulated and inflammatory transcriptional networks that drive astrocyte reactivity and CNS immune amplification. Like DMF, IDMF robustly activates the NRF2 antioxidant pathway, inducing canonical cytoprotective targets (*HMOX1*, *NQO1*, *GCLM*) that enhance glutathione metabolism, neutralize reactive oxygen species, and restore redox homeostasis in astrocytes. This NRF2-driven shift strengthens cellular resilience against oxidative stress, an early and persistent feature of MS pathology.

Beyond this conserved antioxidant profile, transcriptomic analyses reveal that IDMF exerts a more selective and potent suppression of inflammatory gene networks than DMF. IDMF downregulates NF-κB- and IRF1-regulated cytokines and interferon-stimulated genes, including *C3*, *SERPING1*, and *GBP2*, which define the neurotoxic A1 reactive astrocyte phenotype. By attenuating A1-associated pathways, IDMF disrupts astrocyte-mediated propagation of inflammation, complement activation, and neurotoxicity, thereby reducing signals that drive demyelination and neuronal injury.

Benchmarking studies across neuroinflammatory models further demonstrate that IDMF inhibits microglial TNF-α and IL-1β production more effectively than DMF and modulates PBMC cytokine profiles toward a less proinflammatory state. This coordinated repression of CNS and peripheral inflammatory circuits, combined with enhanced NRF2 activation, positions IDMF as a mechanistically refined fumarate derivative capable of delivering dual-action neuroprotection: reinforcement of antioxidant defenses and selective suppression of astrocyte- and microglia-mediated inflammatory cascades. Together, these mechanistic attributes support IDMF as a next-generation fumarate with translational potential in MS, offering improved precision in targeting neuroinflammatory pathways while reducing the liabilities associated with conventional DMF therapy.

### 4.7. Therapeutic Outlook for IDMF

The collective findings from the three studies cited above highlight that IDMF constitutes a promising next-generation fumarate derivative with potential applications in the treatment of neuroinflammatory and autoimmune diseases, including MS and psoriasis, based on IDMF’s ability to:

- Provide targeted modulation of inflammatory pathways: IDMF exerts distinct and selective effects on key inflammatory transcriptional regulators, including NRF2, NF-κB, and IRF1, suggesting a refined immunomodulatory profile compared to DMF. This positions IDMF as a candidate capable of balancing pro- and anti-inflammatory signaling more precisely than existing therapies.
- Attenuate reactive astrocyte phenotypes: IDMF not only mirrors the DMF-induced transcriptomic signature in astrocytes but also more effectively suppresses genes linked to reactive astrocyte phenotypes—key contributors to the pathogenesis of neurodegenerative and demyelinating diseases. This indicates potential for neuroprotection and glial modulation.
- Deliver efficacy across disease models: IDMF exerts broad activity across three preclinical models relevant to both MS and psoriasis, highlighting its multi-tissue efficacy. Importantly, IDMF exhibited superior or comparable activity to DMF in modulating disease-relevant pathways, suggesting translational value in multi-system inflammatory disorders.

### 4.8. Clinical Implications and Future Directions for IDMF

IDMF emerges as a clinically relevant and mechanistically distinct fumarate derivative with several key advantages over conventional DMF:

- Improved safety and tolerability: Its optimized molecular structure and targeted transcriptomic effects may translate to fewer off-target effects and improved tolerability compared to DMF, which is frequently associated with gastrointestinal and flushing-related side effects.
- Dual therapeutic relevance: Demonstrated efficacy in both central nervous system (CNS) and cutaneous models suggests that IDMF may serve as a dual-action therapeutic for comorbid conditions such as multiple sclerosis (MS) and psoriasis, which often co-occur.
- Precision neuroimmunology: Through NRF2 activation and suppression of NF-κB/IRF1 signaling as well as astrocytic reactivity, IDMF represents a step toward precision neuroimmunomodulation, enabling cell type–specific targeting of neuroinflammatory pathways.

In summary, IDMF’s ability to fine-tune transcriptional networks positions it as a next-generation immunotherapeutic candidate across inflammation-driven pathologies, including MS, psoriasis, and potentially other autoimmune or neurodegenerative diseases.

### 4.9. Limitations and Future Clinical Studies

IDMF remains in the exploratory stage for systemic applications, and all current evidence is based solely on preclinical models and gene-expression studies. Although these findings support modulation of key inflammatory and neuroimmune pathways including NRF2 activation, NF-κB/IRF1 suppression, and attenuation of reactive astrocyte phenotypes such mechanisms require confirmation in human tissues and clinical contexts.

A major limitation is the lack of human clinical data, leaving uncertainties regarding systemic safety, pharmacokinetics, dose selection, and tolerability. It is also unclear whether IDMF’s reduced electrophilicity and improved molecular design will translate into fewer systemic adverse effects compared with established fumarates such as DMF and DRF.

Future investigations should begin with early exploratory clinical studies to establish human safety and pharmacokinetic profiles, followed by small proof-of-concept trials in indications such as MS or psoriasis. Integration of biomarker and transcriptomic endpoints will be essential to verify target engagement and mechanistic relevance in vivo. Only after these foundational steps can larger controlled trials determine IDMF’s therapeutic potential and its role among next-generation fumarate-based treatments.

## 5. Concluding Remarks

Isosorbide-based diesters represent a new frontier in dermatologic and neuroinflammatory therapeutics, uniting barrier restoration, anti-inflammatory activity, and molecular precision within a single chemical platform. Among them, IDC and IDL stand out as topical therapies with robust clinical and mechanistic validation, demonstrating the ability to:

- Strengthen epidermal barrier function via activation of genes regulating keratinocyte differentiation, lipid synthesis, and tight junction integrity.
- Upregulate FLG, IVL, and LOR expression at both the transcriptomic (DEG) and protein levels, reinforcing barrier restoration at a molecular scale.
- Enhance hydration through mechanisms beyond passive humectancy, engaging AQP3, AQP9, CD44, and ceramide biosynthesis pathways.
- Suppress inflammation, normalize dysbiosis, reduce corticosteroid reliance, and improve quality of life in AD populations.

Randomized, double-blind clinical trials confirm that IDC + IDL formulations significantly reduce pruritus, disease severity, *S. aureus* colonization, and corticosteroid use while improving sleep quality and extending remission. These findings position IDC and IDL as next-generation, non-steroidal, barrier-restorative therapies with broad applicability across xerosis, photoaged skin, and chronic inflammatory dermatoses such as AD.

Building on these advances, IDMF expands the therapeutic scope to psoriasis and multiple sclerosis. Engineered to preserve the antioxidant and immunomodulatory efficacy of DMF while eliminating its skin-sensitizing liabilities, IDMF demonstrates:

- Potent NRF2 activation coupled with NF-κB/IRF1 suppression, attenuating oxidative stress and inflammatory cascades at their molecular origins.
- Superior inhibition of reactive astrocyte phenotypes in neuroinflammatory models, conferring neuroprotection in MS.
- Dual efficacy across cutaneous (psoriasis) and central nervous system (MS) disease models, underscoring its versatility as a precision immunomodulator.

Preclinical studies consistently reveal that IDMF alleviates psoriasiform lesions, reduces proinflammatory cytokines, and restores redox balance without the sensitization, phototoxicity, or DNA repair inhibition associated with DMF. Transcriptomic profiling further shows broader, more selective pathway modulation than conventional fumarates, highlighting its potential as a next-generation, multi-indication therapy.

In conclusion, IDC and IDL deliver clinically validated, non-steroidal, barrier-restorative therapy for chronic inflammatory and aging skin disorders, while IDMF provides a dual-action, antioxidant and anti-inflammatory platform for both dermatologic and neurologic applications, its systemic use remains exploratory and requires formal clinical validation. Together, these isosorbide-based diesters herald a paradigm shift toward safer, mechanism-driven interventions spanning skin health, chronic inflammatory disorders, and neuroimmunology.

## Figures and Tables

**Table 1:** Overview of Clinical Study Results (IDC + IDL Lotion vs. Vehicle Control).

Parameters	Adult Study (4-Weeks)	Pediatric Study (8-Weeks) (2–17 years old)
EASI Scores Achieved	EASI-75: 57% vs. 25% (*p* = 0.04) at week 4	EASI-50: 81% vs. 56% (*p* = 0.10) * at week 8
Itch Reduction (IVAS)	≥4-point reduction in 66% vs. 44% (*p* = 0.013) at week 4	≥4-point reduction in 43% vs. 13% (*p* = 0.045) at week 8
Topical Corticosteroid (TCS) Use	Reduced by 25% vs. 292% increase (*p* = 0.039) at week 1 Reduced by 46% vs. 220% increase (*p* = 0.08) * at week 2	3.4 g vs. 13.3 g TCS (*p* = 0.12) * at week 8 47% vs. 20% (*p* = 0.097) * never used TCS at week 8
Abundance of *S. aureus*	Reduction vs. no change (*p* = 0.044) at week 4	Reduction vs. elevation (*p*= 0.0000) at week 8
Improvement in Sleep or DLQI Ratings	53% vs. 26% (*p*= 0.02) improvement in group with moderate-to-severe sleeplessness	Significant (*p* = 0.035) vs. insignificant (*p* = 0.35) reduction in DLQI at week 8
Safety	No adverse effects	No adverse effects

* trending toward significance.

## Data Availability

No new data were created or analyzed in this study. Data sharing is not applicable to this article.
